# Crystal structure and nucleic acid binding mode of CPV NSP9: implications for viroplasm in *Reovirales*

**DOI:** 10.1093/nar/gkae803

**Published:** 2024-09-17

**Authors:** Yeda Wang, Hangtian Guo, Yuhao Lu, Wanbin Yang, Tinghan Li, Xiaoyun Ji

**Affiliations:** Department of Infectious Diseases, Nanjing Drum Tower Hospital, State Key Laboratory of Pharmaceutical Biotechnology, School of Life Sciences, Institute of Viruses and Infectious Diseases, Chemistry and Biomedicine Innovation Center (ChemBIC), Institute of Artificial Intelligence Biomedicine, Nanjing University, Nanjing, China; Department of Infectious Diseases, Nanjing Drum Tower Hospital, State Key Laboratory of Pharmaceutical Biotechnology, School of Life Sciences, Institute of Viruses and Infectious Diseases, Chemistry and Biomedicine Innovation Center (ChemBIC), Institute of Artificial Intelligence Biomedicine, Nanjing University, Nanjing, China; Department of Infectious Diseases, Nanjing Drum Tower Hospital, State Key Laboratory of Pharmaceutical Biotechnology, School of Life Sciences, Institute of Viruses and Infectious Diseases, Chemistry and Biomedicine Innovation Center (ChemBIC), Institute of Artificial Intelligence Biomedicine, Nanjing University, Nanjing, China; Department of Infectious Diseases, Nanjing Drum Tower Hospital, State Key Laboratory of Pharmaceutical Biotechnology, School of Life Sciences, Institute of Viruses and Infectious Diseases, Chemistry and Biomedicine Innovation Center (ChemBIC), Institute of Artificial Intelligence Biomedicine, Nanjing University, Nanjing, China; Department of Infectious Diseases, Nanjing Drum Tower Hospital, State Key Laboratory of Pharmaceutical Biotechnology, School of Life Sciences, Institute of Viruses and Infectious Diseases, Chemistry and Biomedicine Innovation Center (ChemBIC), Institute of Artificial Intelligence Biomedicine, Nanjing University, Nanjing, China; Department of Infectious Diseases, Nanjing Drum Tower Hospital, State Key Laboratory of Pharmaceutical Biotechnology, School of Life Sciences, Institute of Viruses and Infectious Diseases, Chemistry and Biomedicine Innovation Center (ChemBIC), Institute of Artificial Intelligence Biomedicine, Nanjing University, Nanjing, China; Engineering Research Center of Protein and Peptide Medicine, Ministry of Education, China

## Abstract

Cytoplasmic polyhedrosis viruses (CPVs), like other members of the order *Reovirales*, produce viroplasms, hubs of viral assembly that shield them from host immunity. Our study investigates the potential role of NSP9, a nucleic acid-binding non-structural protein encoded by CPVs, in viroplasm biogenesis. We determined the crystal structure of the NSP9 core (NSP9^ΔC^), which shows a dimeric organization topologically similar to the P9-1 homodimers of plant reoviruses. The disordered C-terminal region of NSP9 facilitates oligomerization but is dispensable for nucleic acid binding. NSP9 robustly binds to single- and double-stranded nucleic acids, regardless of RNA or DNA origin. Mutagenesis studies further confirmed that the dimeric form of NSP9 is critical for nucleic acid binding due to positively charged residues that form a tunnel during homodimerization. Gel migration assays reveal a unique nucleic acid binding pattern, with the sequential appearance of two distinct complexes dependent on protein concentration. The similar gel migration pattern shared by NSP9 and rotavirus NSP3, coupled with its structural resemblance to P9-1, hints at a potential role in translational regulation or viral genome packaging, which may be linked to viroplasm. This study advances our understanding of viroplasm biogenesis and *Reovirales* replication, providing insights into potential antiviral drug targets.

## Graphical Abstract

**Graphical Abstract:**
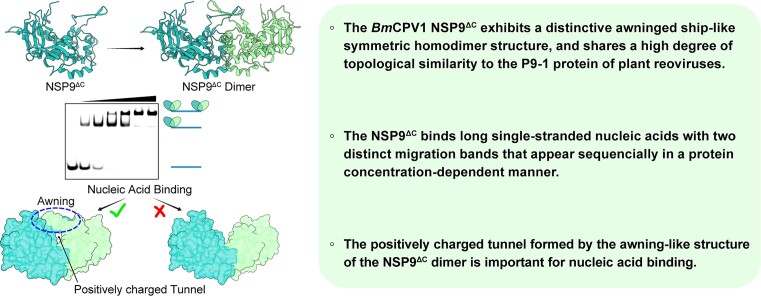

## Introduction

Many viruses require extensive modification of the cellular environment during replication, inducing the formation of distinct intracellular compartments within host cells (1–5). These compartmentalized structures serve as sites for the accumulation of viral proteins, viral nucleic acids, and essential host factors, as well as for virus assembly, and are commonly referred to as viral replication factories, viral inclusions, or viroplasms (6–8). The viral replication factory plays a crucial role in shielding the virus from recognition and attack by the host cell immune system, allowing it to adapt to the complex host cell environment (9–11). Within the viral replication factory, a large number of transcriptional and translational processes take place efficiently (3,12). Different viruses induce the formation of distinct types of viral replication factories, which can be broadly categorized into two groups based on the presence or absence of a membrane structure: membrane-delimited compartments and membrane-less viral inclusions, an electron-dense region observed under the electron microscope, commonly found in viruses of the order *Reovirales* (5,13,14).

Viruses of the order *Reovirales* have icosahedral symmetry but may appear spherical. The capsid is organized as one, two or three concentric layers of capsid proteins, which surround the linear double-stranded RNA (dsRNA) segments of the viral genome. The number of genome segments (9–12) is characteristic of viruses belonging to a single genus (15,16). Viruses classified in the *Reovirales* have a broad host range, infecting mammals, fish, birds, reptiles, arthropods, algae, fungi and plants. Despite the differences between various genera of *Reovirales*, there are remarkable features common to all members of the *Reovirales*, such as large electron-dense viral inclusion bodies or viroplasms, as described above, which have been reported to form during the replication of reoviruses (5,7). Representative viroplasms reported in *Reovirales* include the important pathogens triple-layered rotaviruses (17) and double-layered mammalian orthoreoviruses (MRVs) (18), as well as the single-capsid cypoviruses (CPVs) (19).

Previous studies have shown that phase separation induced by viral biomacromolecules plays a crucial role in the formation of cytoplasmic viral inclusions (9,11). This phenomenon is particularly valuable in the study of membrane-less cytoplasmic viroplasms, such as those of *Reovirales* (20). The viroplasm of the order *Reovirales* consists mainly of virus-encoded non-structural proteins (NSPs). These proteins vary among different viruses within the order *Reovirales*, including σNS and μNS for MRVs (21), NSP2 and NSP5 for rotaviruses (22), NS2 for orbiviruses such as bluetongue virus (BTV) (23), P9-1 for several plant reoviruses such as rice black-streaked dwarf virus (RBSDV), southern rice black-streaked dwarf virus (SRBSDV) and *Mal de Río Cuarto virus* (MRCV) (24–26), and NSP8 and NSP9 for CPV (27). Notably, a recent study indicates that the MRCV P9-1 protein may have a function similar to that of rotavirus NSP2 and orthoreovirus σNS, which are involved in the viral genome packaging process (26). Despite extensive studies of these framework proteins, the precise mechanisms of viroplasm remain elusive. Understanding the key proteins involved not only improves our understanding of the structure of the viroplasm but also helps to elucidate its complex functional mechanisms. CPV is the simplest representative member of the *Reovirales*. It serves as a valuable model to study the viroplasm through the investigation of NSP8 and NSP9. These NSPs are likely to play essential roles within the viroplasm, potentially linking viral protein activity to the highly dynamic processes of viral transcription and translation.

CPV particles contain 10 dsRNA genomic segments encoding 10–11 viral proteins (28). The genome sequence of *Bombyx mori* cypovirus 1 (*Bm*CPV1) has been reported, with identical 5′ (5′ AGUAA- 3′) and 3′ (5′ -GUUAGCC 3′) termini for all 10 segments (29,30). Segments 1, 2, 3, 4, 6 and 7 encode viral core proteins, while segments 5, 8 and 9 produce non-structural proteins. The smallest segment 10, known as the *polyhedrin* gene, encodes a major component of the polyhedra (31). Specifically, segment 8 (S8) encodes the non-structural protein NSP8 (also known as NSP2 or p44) (27,32), which is enriched in the viroplasm and serves as the major scaffolding protein of the viroplasm (27). Segment 9 (S9) encodes another non-structural protein NSP9, which is expressed at an early stage after viral infection (33,34). NSP9 interacts with NSP8 and has nucleic acid binding ability (27,33,34). Apart from these, little is known about NSP9. Questions arise regarding the role of NSP9 when recruited into the viroplasm, the potential implications of its nucleic acid binding activity, and its possible connection to viral transcription or translation. Understanding the structure and function of viroplasm components sheds light on the precise coordination mechanisms underlying viral replication and packaging.

In this study, CPV NSP9 was discovered to oligomerize and exhibit a binding affinity for both DNA and RNA, including both double-stranded (ds) and single-stranded (ss) nucleic acids. Deletion of the C-terminal tail (NSP9^ΔC^) allows us to obtain a homogeneous dimer of NSP9. A high-resolution crystal structure was determined to show the dimeric organization of the protein. A remarkable and conserved nucleic acid binding pattern emerged from gel migration assays (EMSA), revealing two distinct protein-nucleic acid complexes that appeared sequentially in a protein concentration-dependent manner. Structural comparison of NSP9 with plant reovirus protein P9-1 and the similar gel migration pattern observed for both NSP9 and rotavirus NSP3 may suggest some homologous functions in the viroplasm, including viral genome packaging and translation regulation processes.

## Materials and methods

### Plasmids construction

The full-length genes encoding *Bm*CPV1 NSP9 (GenBank: AAC27295.1) and rotavirus_NSP3^1–170^ (GenBank: ADW09100.1) were synthesized by GenScript (Nanjing, China). The fragments of NSP9^ΔC^, NSP9^ΔC^ (Δ9–24), NSP9^ΔC^ (Δ76–117), NSP9^ΔC^ (S18A), NSP9^ΔC^ (K20E) and rotavirus_NSP3^1–170^ were amplified by PCR using KOD-Plus-Neo polymerase (Toyobo, Osaka, Japan), and cloned into the expression vector pMAT9s fused with 6 × His tag and MBP tag followed by the SARS main protease (SARS-M^pro^) cleavage site recognizing the sequence (TTVKLQAGF) upstream of the target proteins. The fragments of NSP9^FL^ fused to the C-terminal StrepII tag were cloned into a pFastBac Dual vector.

### Protein expression and purification

The recombinant plasmids of pMAT9s-NSP9^ΔC^, pMAT9s-NSP9^ΔC^ (Δ9–24), pMAT9s-NSP9^ΔC^ (Δ76–117), pMAT9s-NSP9^ΔC^ (S18A), pMAT9s-NSP9^ΔC^ (K20E) and pMAT9s-rotavirus_NSP3^1–170^ were transformed into *E. coli* BL21 (DE3) Codon Plus cells, which were grown in Terrific Broth (TB) medium supplemented with ampicillin (100 mg/ml) and chloromycetin (34 mg/ml) at 37°C until the OD_600_ reached 0.9. Growth was induced with 0.2 mM isopropyl β-d-thiogalactoside (IPTG) for 18 h at 16°C. Cells were harvested by centrifugation at 3500 rpm (JLA-8.1000, Beckman Coulter, Germany) and resuspended in a buffer containing 20 mM HEPES (pH 7.5), 200 mM NaCl, and 1 mM PMSF. The cell suspension was then lysed by homogenization and clarified by centrifugation at 16 000 rpm (JA-25.50, Beckman Coulter, Germany) at 4°C for 60 min. The recombinant protein was purified on an MBP-Trap HP affinity column (GE Healthcare, USA) and eluted in 20 mM HEPES (pH 7.5), 200 mM NaCl and 10 mM maltose. The MBP-fused protein was digested with SARS-M^pro^ for 30 min at room temperature. The flow through of ion exchange column HiTrap Q HP (Cytiva, USA) was collected, concentrated and loaded onto a HiLoad 16/600 Superdex 75 column (Cytiva, USA) equilibrated with 10 mM HEPES (pH 7.5), 60 mM NaCl. Peak fractions were analyzed by SDS-PAGE and stained with Coomassie blue.

Additionally, a recombinant plasmid of pIEx/Bac1-NSP9^ΔC^ was constructed for transient expression in High Five cells (Invitrogen). The cells were cultured in ESF 921 medium (Expression Systems, LLC) at 27°C at 120 rpm. Transfection was conducted when the cell density reached 4 × 10^6^ cells/ml. Approximately 1 μg of plasmid DNA per 1 × 10^6^ cells was added directly to the cells, followed by the immediate addition of 4 μg of 40 kDa polyethylenimine (PEI40, 1 mg/ml stock solution in H_2_O, Polysciences) per 1 × 10^6^ cells (35). After a cultivation of 6 h, three times the original volume of ESF 921 media was added. Transfected cells were harvested 72 hours post-transfection. The subsequent protein purification method was consistent with that of NSP9^ΔC^ expressed in *E. coli*.

The recombinant baculovirus containing NSP9^FL^ was prepared from *Spodoptera frugiperda* (*Sf*9) cells. 500 ml of *Sf*9 cells infected with P3 recombinant virus at a ratio of 1:500 were harvested 3 days post infection (dpi) by centrifugation at 1500 rpm (JLA-8.1000, Beckman Coulter, Germany) and resuspended in buffer containing 20 mM HEPES (pH 7.5), 200 mM NaCl, 1 × protease inhibitor (Roche, USA), followed by homogenization and clarified by centrifugation at 17 000 rpm (JA-25.50, Beckman Coulter, Germany) at 4°C for 60 min. The recombinant protein fused to the StrepII tag was purified using StrepTactin resin StrepTactin XT-4T (IBA Lifesciences, Germany) and eluted in 20 mM HEPES (pH 7.5), 200 mM NaCl and 2.5 mM d-desthiobiotin (Sigma, USA).

### Protein crystallization and data collection

NSP9^ΔC^ was crystallized using sitting-drop method by mixing 0.2 μl protein solution at a concentration of 4 mg/ml and 0.2 μl precipitation solutions from various commercial sources (NeXtal Tubes Classic Suite Cat No.: 130701, NeXtal Tubes ProComplex Suite Cat No.: 130721, MD Tube Set JCSG-plus Lot No.: ANA1036, MD Tube Set PACT premier Lot No.: ANA1494). High-quality crystals were obtained after 1–2 days at 20°C in the conditions of 0.1 M Bis–Tris (pH 8.0), 20% (w/v) polyethylene glycol (PEG) 4000 or 0.02 M sodium/potassium phosphate, 0.1 M Bis-Tris propane (pH 8.5), 20% (w/v) PEG3350. Crystals were further refined by mixing 1 μl of freshly prepared protein solution at the concentration of 5 mg/ml with 1 μl of the precipitant solution described above. Crystals were cryoprotected with 20% (v/v) glycerol-containing precipitant solution and flash-frozen in liquid nitrogen. Diffraction data were collected at the Shanghai Synchrotron Radiation Facility (SSRF) beamline BL18U1 or BL19U1.

### Structure determination and refinement

Diffraction images were indexed, integrated and scaled using XDS or HKL2000 (36). The search model used for molecular replacement was predicted and generated by Shanghai Zelixir Biotech Company Ltd.. Subsequent iterative refinement with Phenix (37) and manual building in Coot (38) yielded a final structure at 1.80 Å resolution with two homodimers in the asymmetric unit. Data statistics are shown in Table 1.

**Table 1.** X-ray diffraction data collection and refinement statistics

	*Bm*CPV1 NSP9^ΔC^
**Data collection**	
Beamline	SSRF-BL18U1
Wavelength (Å)	0.9792
Space group	*P*2_1_2_1_2_1_
**Cell dimensions**	
*a*, *b*, *c* (Å)	82.43, 83.57, 184.41
α, β, γ (°)	90, 90, 90
Resolution range^a^ (Å)	40.22–1.80 (1.86–1.80)
Unique reflections	117 190 (11 571)
Completeness (%)	98.91 (98.55)
Redundancy	8.8 (9.0)
*I*/σ*I*	13.39 (1.19)
*R*_merge_	0.100 (1.607)
CC_1/2_	0.998 (0.593)
**Refinement**	
Resolution range (Å)	41.21–1.80 (1.86–1.80)
No. reflections (working/test)	117149/11550
*R*_work_/*R*_free_	0.187/0.204
**Number of atoms**	
Macromolecules	7685
Water	645
***B*-factors**	
Macromolecules	44.23
Water	44.88
**R.m.s. deviations**	
Bond lengths (Å)	0.01
Bond angles (°)	1.02
**Ramachandran plot**	
Favored (%)	99.28
Allowed (%)	0.72
Outliers (%)	0.00

Single crystal was used for data collection and structure determination.

^a^Numbers in the brackets are for the highest resolution shell.

### Immune fluorescence assay (IFA)

The recombinant baculovirus co-expressing NSP8^FL^ and NSP9^FL^ was prepared from *Sf*9 cells. The floating *Sf*9 cells were seeded on a 35 mm confocal dish for 15 min and washed with PBS buffer 60 h post infection (hpi) with P3 virus. They were then fixed with methanol for 10 min, washed three times with PBS buffer, and blocked with 2% BSA in PBST buffer. Rabbit anti-His polyclonal antibody (ABclonal, China, Cat. No.: AE068) and mouse anti-StrepII monoclonal antibody (ABclonal, China, Cat. No.: AE066) were utilized for a 1.5 h incubation. Cells were then washed three times for 5 min each with PBST, followed by incubation with Alexa Fluor® 488-conjugated goat anti-rabbit antibody (ABclonal, China, Cat. No.: AS053) and Alexa Fluor® 647-conjugated goat anti-mouse antibody (ABclonal, China, Cat. No.: AS059) for 40 min. Subsequently, the cells were stained with 4,6-diamidino-2-phenylindole (DAPI) for 10 min. Images were captured using a Zeiss LSM 980 scanning confocal microscope and processed with ZEN software (Carl Zeiss, Germany).

### Electrophoretic mobility shift assay (EMSA)

The nucleic acid binding ability of NSP9^ΔC^ and rotavirus_NSP3^1-170^ was detected by EMSA. The oligonucleotides used in the EMSA assay were synthesized with or without a 5′ carboxyfluorescein (FAM) label from GenScript Co., Ltd. Double-stranded nucleic acids used in this assay were annealed with an equimolar amount of both forward and reverse strands. The shorter (15 nt or 15 bp) sequence was AGTAAGCTGTTAGCC (DNA) or AGUAAGCUGUUAGCC (RNA). The longer (30 nt or 30 bp) sequence was AGTAAATCCCAGGCGCTCGGGTCGTTAGCC (DNA) or AGUAAAUCCCAGGCGCUCGGGUCGUUAGCC (RNA). The sequence for the 40 nt DNA was AGTAAATCCCAGGCGTAAACCGAATATCGCTACATTCGCA. The sequences of the RNA used for rotavirus NSP3 detection were GGCUUUUAAACGAAGGAUGUGACC and 24 nt poly(A). 3 μM DNAs or RNAs were mixed with increasing amounts of protein in a reaction buffer (10 mM HEPES pH 7.5 and 60 mM NaCl), then loaded onto a 6% native PAGE with 5 × loading buffer (150 mM NaCl, 25 mM Tris–HCl pH7.5, 0.2 mg/ml BSA, 50% glycerol) and electrophoresed in running buffer containing 0.5 × Tris–borate–EDTA (TBE) under an electric field of 10 V/cm for approximately 30 min on ice. The gels were visualized and analyzed using the green fluorescent lamp module of a Tanon-5100 Imaging System (Tanon Science & Technology, China) and its Tanon Image software or Coomassie blue staining.

### Analytical ultracentrifugation (AUC)

The sedimentation velocity (SV)-AUC experiment was performed in a Beckman Coulter XL-I analytical ultracentrifuge using a two-channel centerpiece equipped with an AN-60 Ti rotor (Beckman Coulter, Germany). The protein solution and control group were each loaded into 120-mm double-sector aluminum centerpieces and run at a rotor speed of 40 000 rpm under vacuum. Data were collected at 16°C, and the absorbance of NSP9^ΔC^ was collected at 280 nm. SV-AUC data were analyzed using the SEDFIT program and fitted to a continuous *c*(*s*) distribution model to determine the sedimentation coefficient and molecular mass of each peak (39). Data were visualized using GraphPad Prism 8 software (https://www.graphpad.com/).

### Negative staining

NSP9^FL^ was evaluated by negative staining. 5 μl of purified samples (0.02 mg/ml) were applied to the glow-discharged copper grids (Electron Microscopy China) for 1 min and then blotted with filter paper. Then, the grids were negatively stained with 2% (w/v) uranyl acetate for 1 min, gently blotted and air dried. Images were captured with a Talos L120C TEM (Thermo Fisher Scientific) equipped with a CCD camera.

### Size-exclusion chromatography (SEC) analysis

SEC analysis of purified NSP9^FL^ was carried out on a Superose 6 Increase 10/300 column (GE Healthcare, USA). SEC analysis of purified NSP9^ΔC^, NSP9^ΔC^ (Δ9–24), NSP9^ΔC^ (Δ76–116), NSP9^ΔC^-DNA complex and apo formed DNA on a Superdex 200 Increase 10/300 column (GE Healthcare, USA). SEC analysis of purified rotavirus_NSP3^1-170^ was carried out on a HiLoad 16/600 Superdex 75 pg column (GE Healthcare, USA). All columns were pre-equilibrated with buffer containing 10 mM HEPES (pH 7.5) and 100 mM NaCl. Elution volumes for molecular weight markers were used for comparison. Data were visualized using GraphPad Prism 8 software.

## Results

### NSP9 is potentially involved in viroplasm via interaction with NSP8 in insect cells

**Figure 1.**
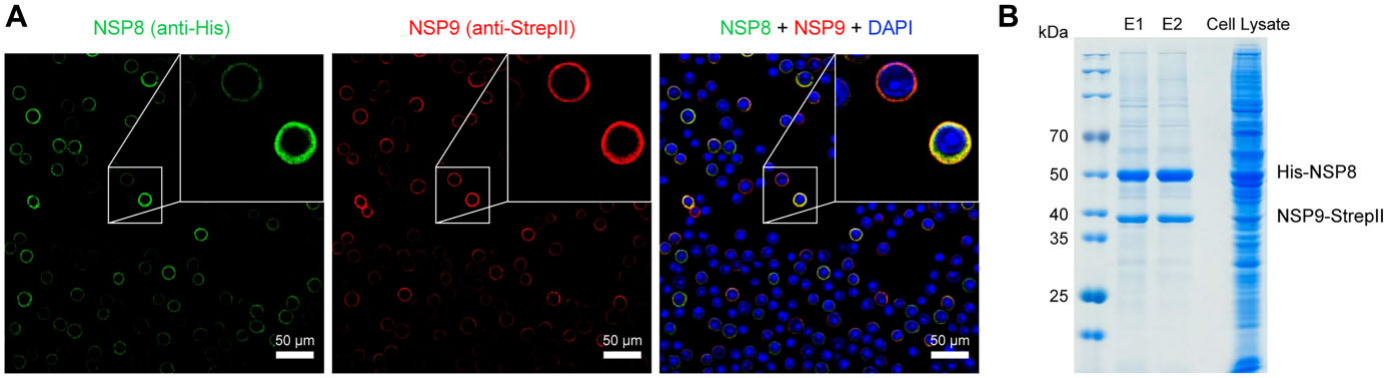
The interaction between NSP9 and the viroplasm scaffold protein NSP8. (**A**) Overexpression of His tag fused NSP8 and StrepII tag fused NSP9 in *Sf*9 cells with recombinant baculovirus infection, immunofluorescence assay showed that the colocalization of NSP9 (red, 647 nm stimulation) and NSP8 (green, 488 nm stimulation) and distributed in the cytoplasm. (**B**) Co-expression of His tag fused NSP8 and StrepII tag fused NSP9 with recombinant baculovirus infection in *Sf*9 cells and purified with StrepTactin beads. Two sequential elution products (E1 and E2), as well as the whole-cell lysate sample, were detected by SDS-PAGE.

Previous studies have identified NSP8 as a key component of the viroplasm in CPV from *Dendrolimus punctatus*, demonstrating extensive interactions with both structural and non-structural proteins (27). To investigate this complex network, we examined the interactions between NSP9 and the viroplasm scaffolding protein NSP8. A recombinant baculovirus for the expression of both NSP8 and NSP9 proteins of *Bm*CPV1 in *Sf*9 cells was generated using a recombinant co-expression plasmid. Confocal microscopy results obtained through immunofluorescence staining indicated that NSP9 (StrepII-tagged) and NSP8 (His-tagged) were both distributed in the cytoplasm, with their fluorescence signals exhibiting partial overlaps (Figure 1A). Furthermore, StrepII pull-down assays further confirmed the close association between NSP9 and NSP8. The His-tagged NSP8 was co-purified with the StrepII-tagged NSP9 using StrepTactin resin (Figure 1B). Given that NSP9 can also bind to nucleic acid, these results suggest that NSP9 interacts with the viroplasm scaffold protein NSP8 and may be a component of the viroplasm.

### The C-terminal tail mediates the oligomerization of dimerized NSP9

**Figure 2.**
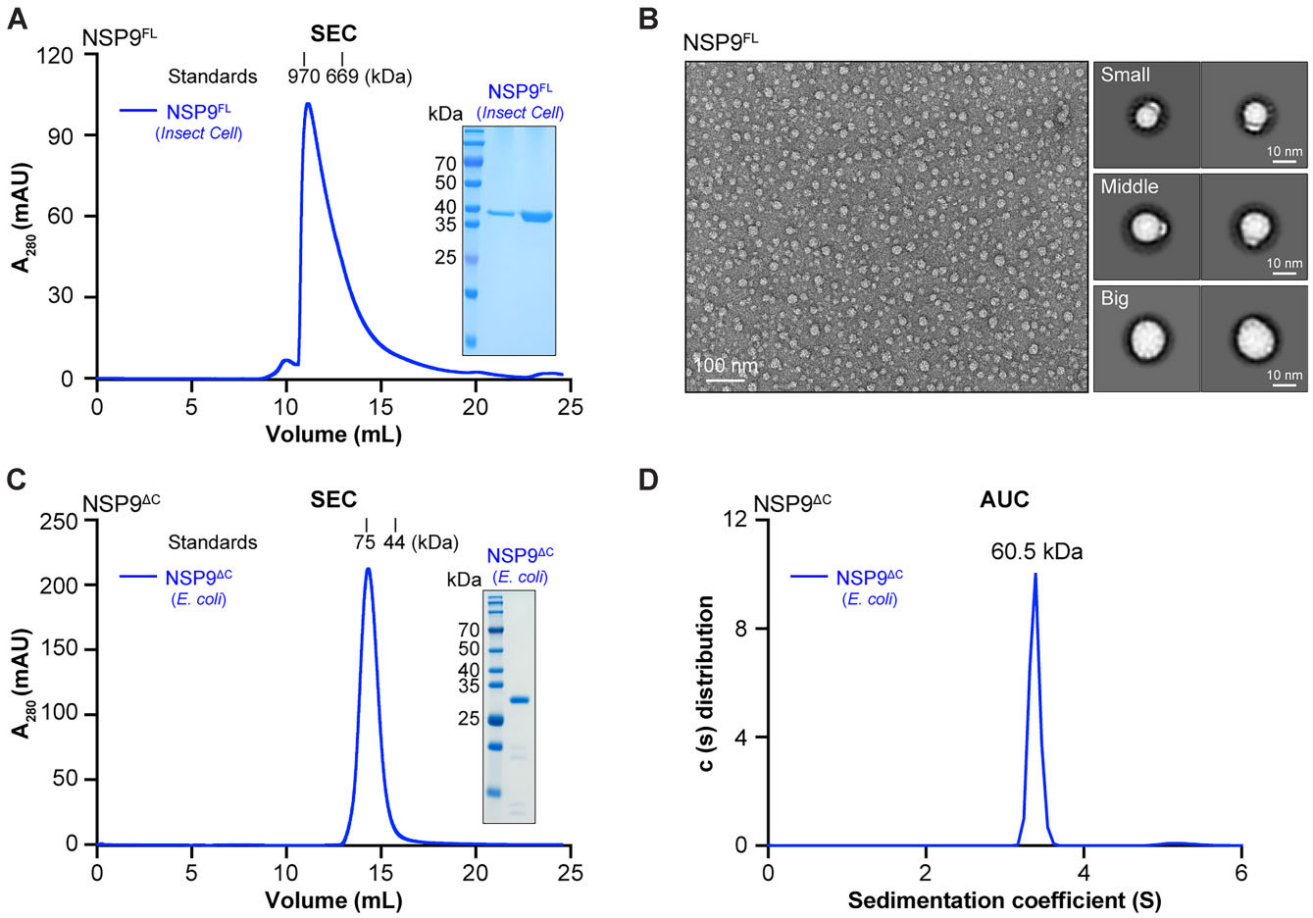
Analysis of NSP9^FL^ and NSP9^ΔC^ in solution revealed that the C-terminal region induced the assembly of high molecular weight multimers of NSP9^FL^, while NSP9^ΔC^ exhibited a dimeric arrangement. (**A**) SEC analysis of purified NSP9^FL^ proteins expressed in *Sf*9 cells using a Superose 6 Increase 10/300 column and the SDS-PAGE analysis of peak fractions of interest. (**B**) A representative micrograph of negatively stained NSP9^FL^, followed by a 2D classification of auto-picked particles, reveals a mixture of particles of different sizes. The scale bar was 100 nm. (**C**) SEC analysis of purified NSP9^ΔC^ proteins expressed in *E. coli* using a Superdex 200 Increase 10/300 column and the SDS-PAGE analysis of peak fractions of interest. (**D**) SV-AUC analysis of the molecular weight of the NSP9^ΔC^ dimer in solution. Experiments were performed twice with equivalent results.

Full-length NSP9 (NSP9^FL^) from *Bm*CPV1 fused with a C-terminal StrepII tag was expressed and purified from *Sf9* cells. Size exclusion chromatography (SEC) confirmed the presence of a heterogeneous polymer of approximately 900 kDa (Figure 2A). The negative staining electron microscopy images, processed using automatic particle picking and 2D classification, revealed a heterogeneous spherical assembly with a diverse diameter of 10–20 nm and an estimated molecular weight of 800–6000 kDa (40) (Figure 2B). Attempts to crystallize the full-length NSP9 protein were unsuccessful, probably due to its propensity to multimerize into heterogeneous complexes.

The NSP9 C-terminus exists as a highly intrinsic disordered region (IDR), as predicted by IUPred3A (41), and two potential stable fragments have been identified (Supplementary Figure S1A). The restriction protease lysate assay, with elastase and V8 protease digestion, yielded fragments with a theoretical molecular weight of approximately 30–32 kDa (Supplementary Figure S1B and C). Accordingly, we expressed a truncated NSP9 (NSP9^ΔC^, lacking residues 287–320) from *Bm*CPV1 in *E. coli* as an N-terminally MBP-tagged protein. NSP9^ΔC^ (1–286 amino acid residues) was purified by Ni-NTA affinity chromatography, and its purity was confirmed by a single band on SDS-PAGE and Coomassie Brilliant Blue staining (Figure 2C). The purified NSP9^ΔC^ existed as a dimer in solution, as confirmed by SEC and analytical ultracentrifugation (AUC), with a precise molar mass of 60.5 kDa (Figure 2D). Importantly, these results were also consistent with the SEC profile obtained for NSP9^ΔC^ expressed in insect cells (Supplementary Figure S2), indicating that NSP9^FL^ behaves as a higher-order oligomer, while the NSP9^ΔC^ construct remains a homogeneous dimeric state. These findings align with previous observations in P9-1 proteins of fijiviruses RBSDV, SRBSDV and MRCV, where the C-arm is essential for octamer formation but not for dimer assembly (24–26). Thus, the results emphasize the crucial role of the C-terminal tail in *Bm*CPV1 NSP9 as it serves as an important structural component that initiates the assembly of a heterogeneous spherical polymer.

### The crystal structure of *Bm*CPV1 NSP9^ΔC^ shows a dimeric conformation

The purified NSP9^ΔC^ was crystallized in an orthorhombic space group *P*2_1_2_1_2_1_, with unit cell dimensions of *a* = 82.4 Å, *b* = 83.6 Å and *c* = 184.4 Å (Table 1). The crystal structure was solved using the molecular replacement method with the AI-predicted atomic coordinates of *Bm*CPV1 NSP9 as a search model. In the asymmetric unit, four molecules of NSP9^ΔC^ were identified and organized into two virtually identical dimers, with a Root Mean Square Deviation (RMSD) of 0.6 Å. The final 2*mF_o_-DF_c_* electron density map exhibited consistency, with no chain breaks for most of the protein backbone. Notably, the missing regions included the C-terminus (residues 285–286) and a flexible loop (residues 85 to 110). The final refined model achieved a high resolution of 1.80 Å, exhibiting favorable stereochemistry parameters and refinement statistics (*R*_work_ = 0.18 and *R*_free_ = 0.20).

**Figure 3.**
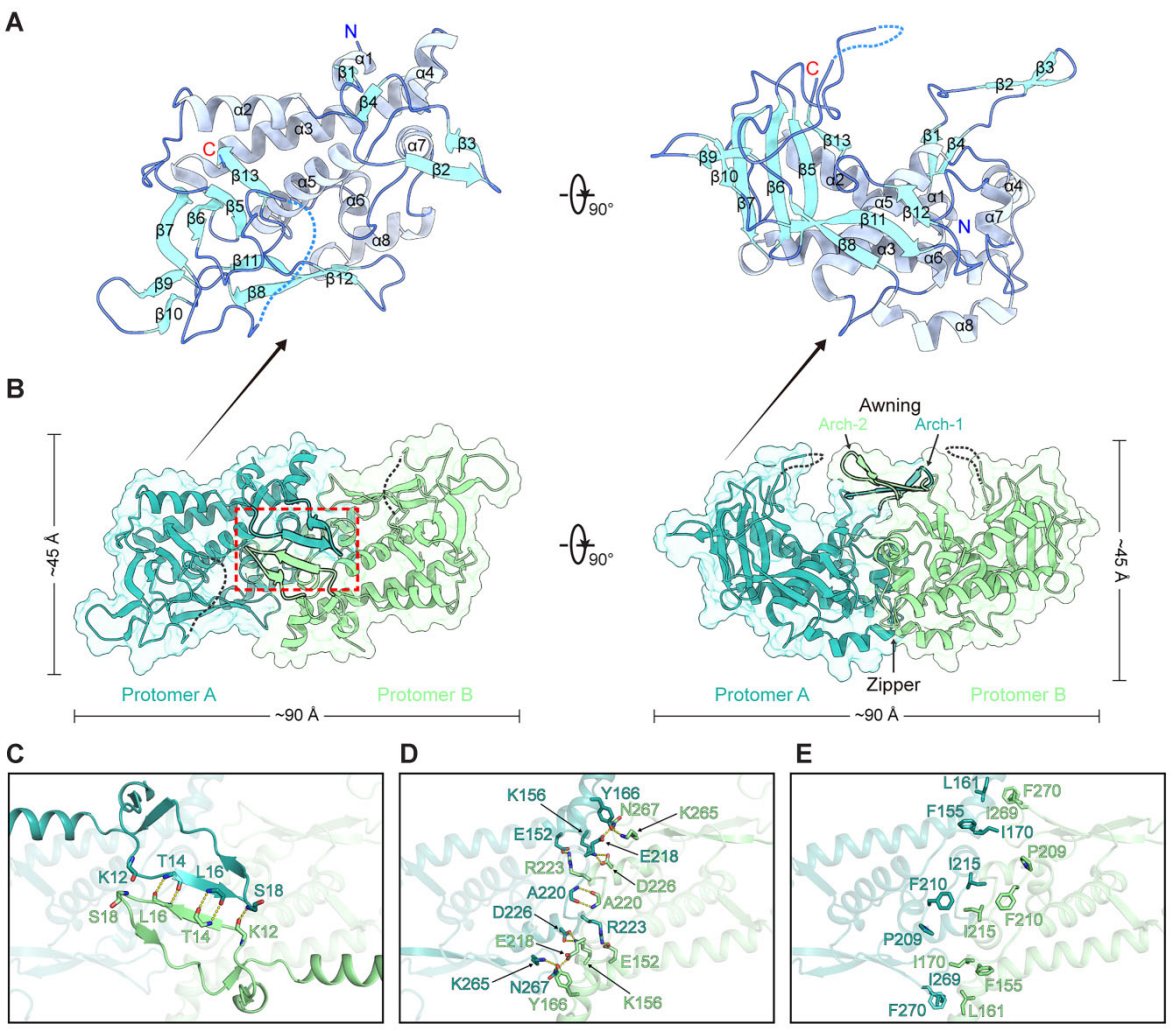
The crystal structure of NSP9^ΔC^ and detailed interactions in the homodimer interface. (**A**) The monomer structure of NSP9^ΔC^ in two orthogonal views, shown as cartoon representations. The secondary structure elements are labeled and colored by type: α-helices, light purple; β-strands, sky blue; loops, royal blue. Structure elements are organized as follows: α1 (residues 1–4), α2 (32–43), α3 (133–155), α4 (163–171), α5 (175–191), α6 (200–207), α7 (213–218), α8 (224–234); β1 (5–7), β2 (14–17), β3 (20–22), β4 (26–29), β5 (59–63), β6 (58–73), β7 (122–128), β8 (238–242), β9 (245–247), β10 (252–254), β11 (257–264), β12 (271–274) and β13 (280–283). The N and C termini are labeled. (**B**) The homodimer structure of NSP9^ΔC^ in two orthogonal views, shown as both cartoon and surface representations. Protomers A and B in NSP9^ΔC^ homodimer are colored sea green and pale green, respectively. The awning-like structure is boxed with red dashed lines. (**C**) The detailed dimeric interactions in the awning region of the homodimer between two NSP9^ΔC^ protomers. Potential hydrogen bonds are represented as yellow dashed lines and the related residues are shown as sticks. (D, E) The detailed dimeric interactions in the zipper region of the homodimer between two NSP9^ΔC^ protomers. Potential hydrogen bonds and salt bridges are represented as yellow dashed lines and the related residues are shown as sticks (**D**), and potential hydrophobic interacting residues are shown as sticks (**E**).

The overall structure of the NSP9^ΔC^ subunit revealed a compact architecture composed of 8 α-helices (α1 to α8) and 13 β-strands (β1 to β13) (Figure 3A). The longest α-helix α3 (residues 132–156) spans ∼36 Å across the entire polypeptide fold, enclosed by 7 other α-helices to form a helical bundle. The subunit structure comprises five sets of antiparallel β-strand sheet, including β1↑β4↓ (β-sheet I), β2↑β3↓ (β-sheet II), β5↓β6↑β7↓β13↑ (β-sheet III), β8↑β11↓β12↑ (β-sheet IV) and β9↑β10↓ (β-sheet V), respectively. Notably, β-sheet I tightly integrates into the helical bundle, surrounded by α-helices α1 to α5. In contrast, β-sheet II extends outward, forming an exposed arch-like structure (Figure 3A). β-sheets III to V align along the same side of α-helix α3, with β-sheet III exhibiting an almost perpendicular orientation to β-sheets IV and V (Figure 3A). While most β-sheets typically feature adjacent strands connected by well-defined β-hairpins or longer loops, the loop between β6 and β7 (residues 74–121) displays only partial continuity in the electron density map. This aligns with the IDR prediction in this region based on the NSP9 amino acid sequence (Supplementary Figure S1A), indicating significant local flexibility.

The NSP9^ΔC^ homodimer exhibits a distinctive awninged ship-like structure (Figure 3B). Two NSP9^ΔC^ protomers adopt highly symmetric conformations, featuring a 2-fold rotational symmetry axis perpendicular to the ship's hull (the main body of the homodimer). To investigate the key components contributing to homodimer formation, we conducted a comparative analysis of the interactions within the dimer interface. The dimer interface can be divided into two regions. The first region involves an antiparallel β-sheet formed by β2 from both protomers, forming five pairs of hydrogen bonds through the backbone (Figure 3C). This intermolecular β-sheet, combined with β3 from both protomers, further forms a distinctive awning-like structure comprising four antiparallel β-strands, thereby providing enhanced stability to the homodimer (Figure 3B). Interestingly, in our crystal structure, K12 on protomer A (K12^A^) fails to form a robust hydrogen bond with S18 on protomer B (S18^B^) (Figure 3C). This deficiency can be attributed to the larger distance (∼ 4.74 Å) between the carboxyl oxygen atom of K12^A^ and the amino nitrogen atom of S18^B^, compared to the more closely positioned counterparts S18^A^ and K12^B^, which maintain a proper distance of 3.08 Å (Figure 3C). This result illustrates that while the homodimer has a high degree of global 2-fold symmetry, slight conformational differences exist locally, particularly within the flexible regions. The second region involves 40 interface residues from each protomer (15.4% of the total residues), which are included in the α-helices α3, α4, α7 and α8, the loops α3–α4, α6–α7 and α7–α8, and the hairpin β11–β12. Residues E152 (α3), K156 (loop α3–α4), Y166 (α4), E218, A220, R223 (loop α7–α8), D226 (α8), K265 and N267 (hairpin β11–β12) form hydrogen bond or salt bridge interactions (Figure 3D), while hydrophobic residues F155 (α3), L161 (loop α3–α4), I170 (α4), P209, F210 (α6–α7), I215 (α7), I269 and F270 (hairpin β11–β12) form extensive zipper-like interactions with the helical bundle (Figure 3B and E).

### The structure of NSP9 shares conserved features with other non-structural proteins within the *Reovirales*

**Figure 4.**
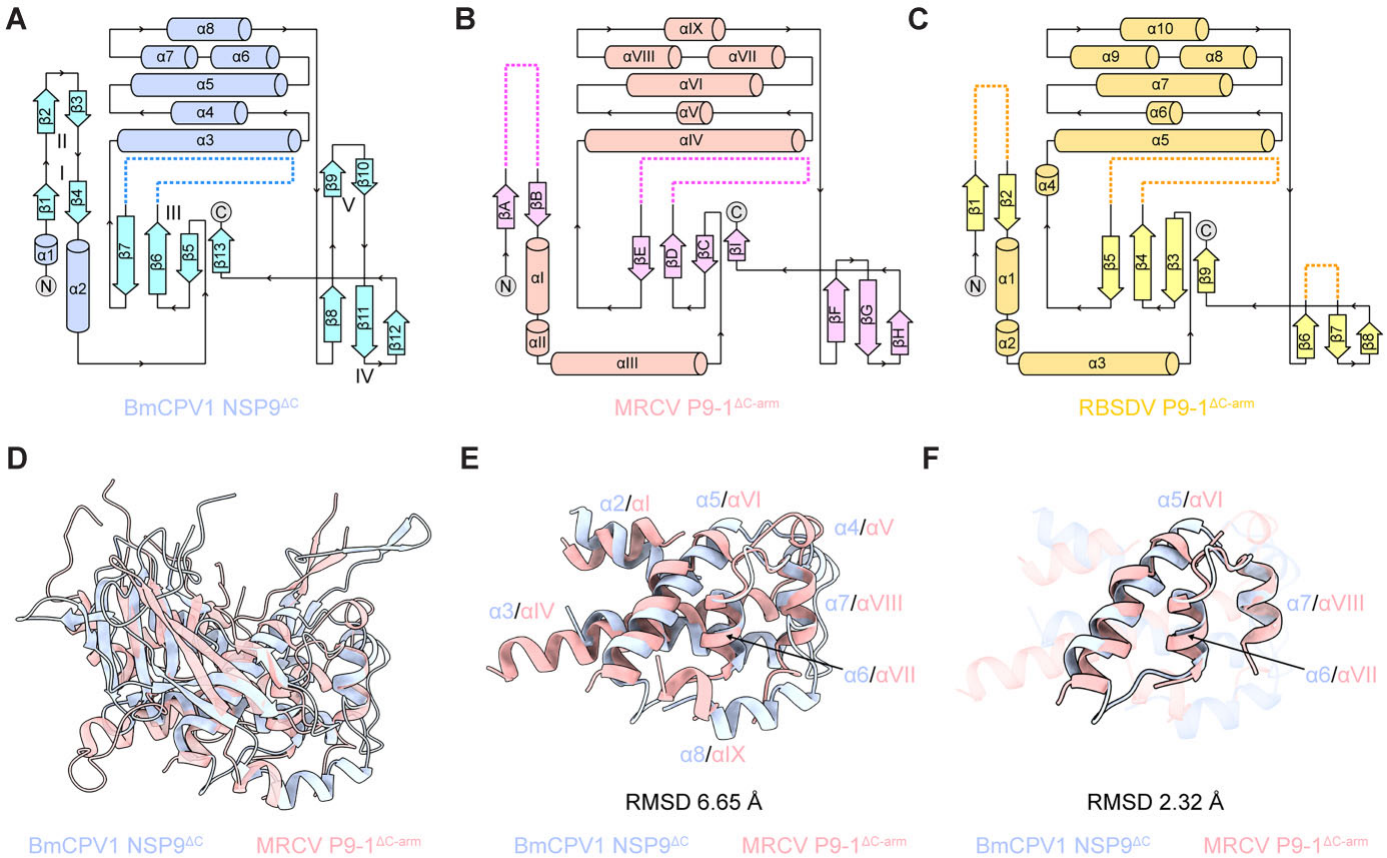
Structural comparison between NSP9^ΔC^ and P9-1 proteins of MRCV and RBSDV. (A–C) Topological structure representations of *Bm*CPV1 NSP9^ΔC^ (**A**), MRCV P9-1^ΔC-arm^ (**B**) and RBSDV P9-1^ΔC-arm^ (**C**), respectively. The spatial distribution characteristics of their secondary structures reveal a remarkably high degree of similarity. (D–F) Structure alignment between NSP9^ΔC^ and MRCV P9-1^ΔC-arm^ of the entire molecule (**D**), the helical bundle (**E**) and the core helices regions (**F**). The NSP9^ΔC^ is colored light purple and the MRCV P9-1^ΔC-arm^ is colored salmon, respectively.

We performed a structure homology search using DALI, a protein structure comparison server (http://ekhidna2.biocenter.helsinki.fi/dali/) (42) and identified hardly any NSP9 homologous structures, except the P9-1 from three plant reoviruses, RBSDV (PDB ID: 3VJJ) (24), SRBSDV (PDB ID: 5EFT) (25) and MRCV (PDB ID: 6UCT) (26). These three counterparts share a substantial sequence identity of over 64% and exhibit high structural similarities with each other (26). The P9-1 proteins from both MRCV and RBSDV have been reported to be localized within the viroplasms of plant and insect hosts (43,44). When expressed alone, it engages in self-interaction, resulting in the formation of cytoplasmic viroplasm-like structures (44–46). Despite significant conformational differences between NSP9^ΔC^ and P9-1, a detailed comparison of their topological structures reveals a remarkable degree of similarity, especially in the spatial distribution characteristics of their secondary structures (Figure 4A–C). Compared to NSP9^ΔC^, P9-1 features an additional α-helix, αIII, positioned between the second α-helix and the subsequent β-strand sheet (Figure 4B and C). Nevertheless, the spatial arrangement of the helical bundles in both proteins remains remarkably similar. Calculating the RMSD between NSP9^ΔC^ and MRCV P9-1^ΔC-arm^ across their entire structures might be meaningless due to the high divergence in their primary sequence (Figure 4D). However, focusing on the core helical bundle alignment yields an RMSD of 6.65 Å (Figure 4E). Furthermore, focusing on three consecutive α-helices (α5 to α7 in NSP9 and αVI to αVII in P9-1) close to the dimer interface reveals an even more similarity, with a minimal RMSD of only 2.32 Å (Figure 4F).

In contrast, the spatial discrepancy of β-sheets between the NSP9 and P9-1 was somewhat more variable. While the arrangement of three β-strand sheets (β-sheets I, III and IV of NSP9) was generally consistent, maintaining a constant number of strands, NSP9 exhibited two additional β-strand sheets, β-sheet II and β-sheet V (Figure 4A). Notably, the region corresponding to β-sheet II in both MRCV P9-1 and RBSDV P9-1 could not be precisely reconstructed due to the poor electron density (Figure 4B and C). Moreover, in MRCV P9-1, β-sheet V was substituted by a hairpin connecting a pair of extended antiparallel β-strands (βF and βG), whereas in RBSDV P9-1, this region remained undefined (26). Interestingly, the NSP9^ΔC^ crystal structure revealed four molecules within the asymmetric unit, arranged in two pairs of noncrystallographic homodimers (Supplementary Figure S3A and B). In the second homodimer, formed by protomers C and D, the amino acids corresponding to β-sheet II also lack electron density information (Supplementary Figure S3C and D). This observation suggests a high degree of dynamics in the region between β1 and β4, highlighting potential structural conservation throughout the order *Reovirales*.

The dimerization interaction of NSP9^ΔC^ involves an interface area of approximately 1536 Å^2^ (11.5% of the total solvent-accessible surface per protomer) between protomers A and B, and approximately 1177 Å^2^ (9.6% of the total solvent-accessible surface per protomer) between protomers C and D due to the lack of the awning-like β-sheet II pairs, both larger than the homodimer interaction of MRCV P9-1 according to the PDBePISA server (26,47). This indicates that NSP9^ΔC^ forms a more tightly packed homodimer with more extensive intermolecular interactions.

### NSP9 protein binds preferentially to single-stranded rather than double-stranded nucleic acids

**Figure 5.**
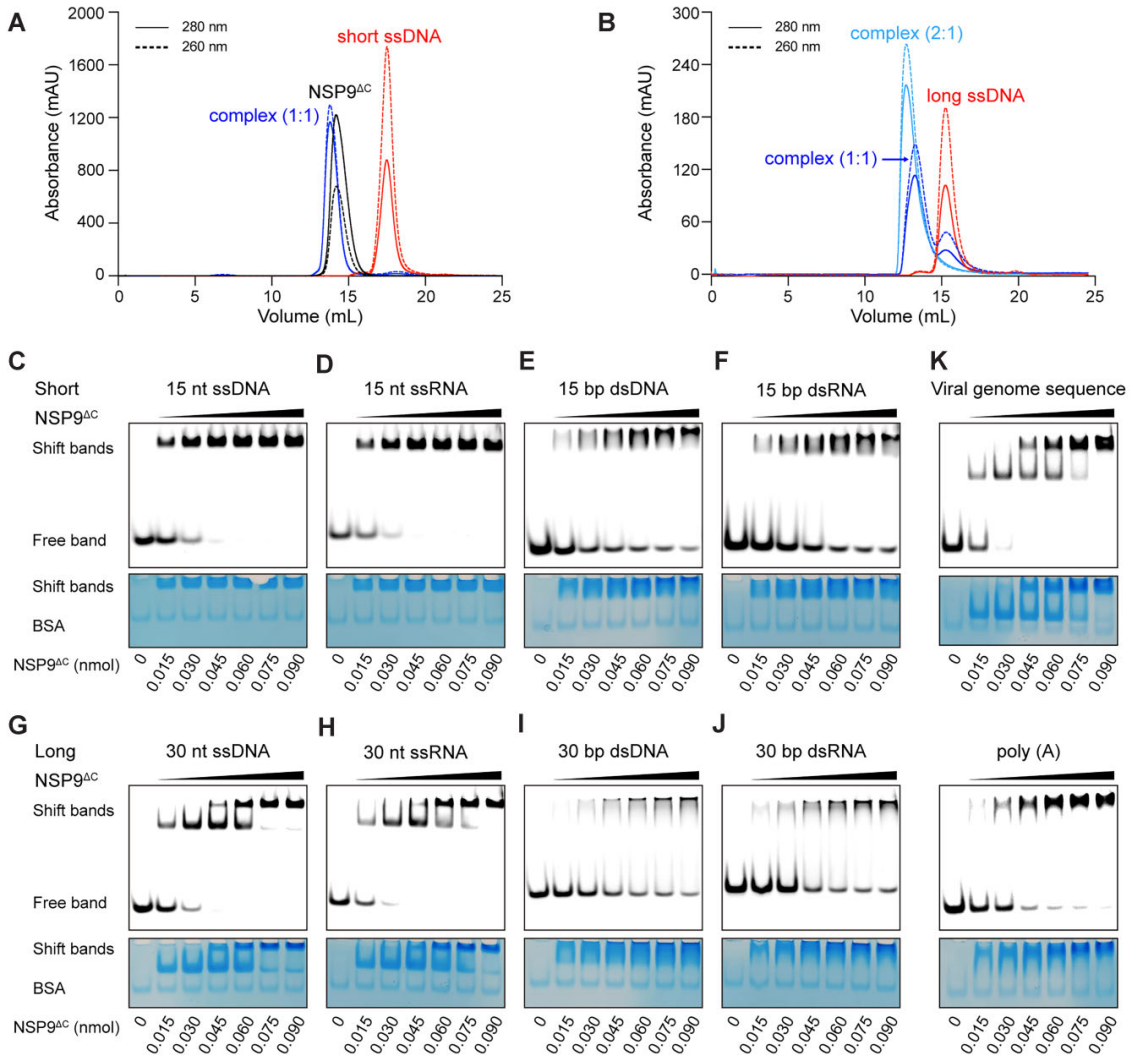
The nucleic acid binding ability of NSP9^ΔC^ and the potential for the formation of two distinct complexes in a protein concentration-dependent manner. (A, B) The complex of NSP9^ΔC^ and short (**A**) or long (**B**) ssDNA was detected by SEC using a Superdex 200 Increase 10/300 column. (C–F) EMSA results of NSP9^ΔC^ with 0.03 nmol 5′ 6-FAM labeled 15 nt ssDNA, 15 nt ssRNA, 15 bp dsDNA, and 15 bp dsRNA, respectively. The amount of protein used in each group is indicated under the gel. (G–J) EMSA results of NSP9^ΔC^ with 0.03 nmol 5′ 6-FAM labeled 30 nt ssDNA, 30 nt ssRNA, 30 bp dsDNA, and 30 bp dsRNA, respectively. The amount of protein used in each group was indicated under the gel. Two different shift bands were sequentially detected when binding to 30 nt single-stranded nucleic acids in an NSP9^ΔC^ concentration-dependent manner. (**K**) EMSA results showed different migration patterns between single-stranded viral genome sequences and the same length of poly(**A**), suggesting that the novel nucleic acid binding pattern exhibited a sequence-dependent manner.

It has been reported that NSP9 can bind to poly(I:C) and viral dsRNA genome (33,34), but the specific characteristics of this binding remain unexplored. First, we performed analytical SEC to assess the oligomerization state of purified NSP9^ΔC^ mixed with synthetic 15 nt DNA. The resulting mixture exhibited a slight shift in elution volume compared to the apo NSP9^ΔC^, indicating that NSP9^ΔC^ can form complexes with DNA while maintaining the dimeric arrangement observed in the apo NSP9^ΔC^ dimer (Figure 5A and B). To evaluate the nucleic acid binding ability of NSP9^ΔC^, we conducted EMSA to characterize the binding patterns between NSP9^ΔC^ and various nucleic acid substrates, including 15 nt ssDNA, 15 nt ssRNA,15 bp dsDNA, 15 bp dsRNA, 30 nt ssDNA, 30 nt ssRNA, 30 bp dsDNA, and 30 bp dsRNA, respectively. A clear shift band was observed in the case of 15 nt ssDNA with increasing NSP9^ΔC^ concentration, the EMSA results also indicating a 1:1 binding molar ratio between NSP9^ΔC^ dimer and 15 nt ssDNA (Figure 5C). Notably, no significant difference was observed in the binding affinity of NSP9^ΔC^ for DNA (Figure 5C) and RNA (Figure 5D). Similar analyses were performed for 15 bp dsDNA (Figure 5E), 15 bp dsRNA (Figure 5F), 30 nt ssDNA (Figure 5G), 30 nt ssRNA (Figure 5H), 30 bp dsDNA (Figure 5I), and 30 bp dsRNA (Figure 5J). The results clearly demonstrate that NSP9^ΔC^ exhibits binding affinity for both DNA and RNA, and it can interact with both single-stranded and double-stranded nucleic acids.

Interestingly, EMSA results suggest a preference of NSP9^ΔC^ for binding to single-stranded nucleic acids. In particular, for longer single-stranded nucleic acids (30 nt), a sequential appearance of two different shift bands was observed in a protein concentration-dependent manner. The molar ratio of the NSP9^ΔC^ dimer binding to nucleic acids was 2:1, indicating that one nucleic acid strand can sequentially bind two NSP9^ΔC^ dimers as the protein concentration increases (Figure 5G and H), which is also consistent with the SEC analysis (Figure 5B). This intriguing binding behavior can only be observed when NSP9^ΔC^ interacts with single-stranded nucleic acids. Double-stranded nucleic acids, whether DNA or RNA, do not exhibit the consecutive appearance of two shift bands (Figure 5I and J), suggesting that the binding mechanism of NSP9 to double-stranded nucleic acids may be different from that of single-stranded-nucleic acids. The presence of two separate shift bands potentially indicates the presence of two distinct complexes. This binding pattern becomes evident when comparing the different migration patterns of certain viral genome sequences compared to poly(A) nucleic acid of equivalent length (Figure 5K).

### Positively charged amino acids among the awning-like structure of NSP9 dimer are important for nucleic acid binding

**Figure 6.**
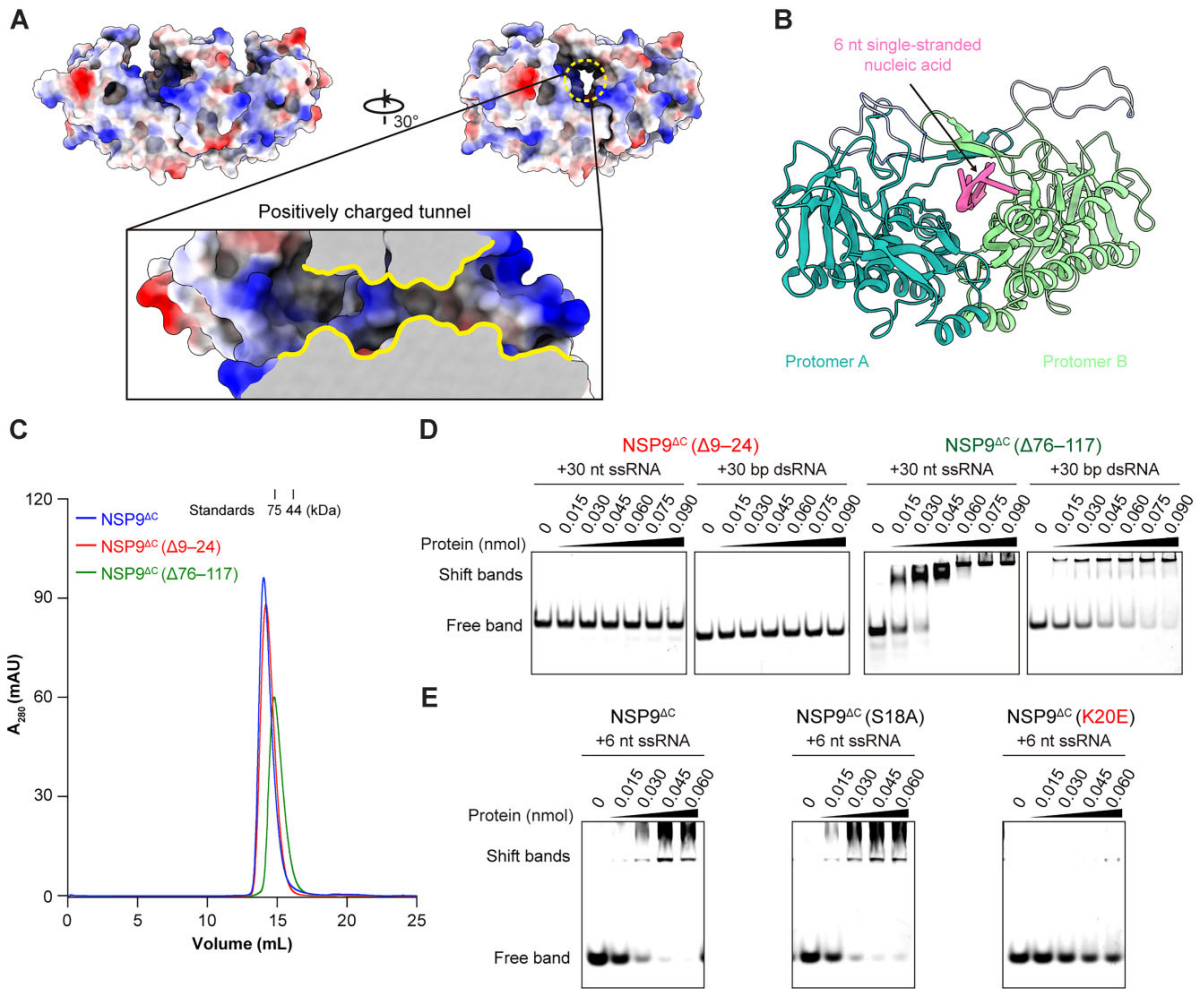
The complex of NSP9^ΔC^ and single-stranded nucleic acid predicted by docking analysis and mutagenesis verification. (**A**) The electrostatic surface of NSP9^ΔC^ dimer. The red color indicates the presence of negatively charged residues, whereas the blue color indicates the presence of positively charged residues. A cut-out view of the positively charged tunnel is highlighted with yellow lines. (**B**) The docking analysis of a 6 nt single-stranded nucleic acid with the NSP9^ΔC^ dimer using the ZDOCK server (https://zdock.wenglab.org/). The 6 nt ssDNA model was partially extracted from PDB ID: 5N8S. (**C**) SEC analysis of purified NSP9^ΔC^ (blue), NSP9^ΔC^ (Δ9–24) (red) and NSP9^ΔC^ (Δ76–116) (green) proteins using a Superdex 200 Increase 10/300 column. (**D**) Comparison of EMSA results of NSP9^ΔC^ (Δ9–24) and NSP9^ΔC^ (Δ76–116) with either 30 nt ssRNA or dsRNA revealed that the 9^th^–24^th^ amino acids are the key nucleic acid binding region. (**E**) Comparison of EMSA results of NSP9^ΔC^, NSP9^ΔC^ (S18A) and NSP9^ΔC^ (K20E) with 6 nt ssRNA revealed that K20 plays a key role in nucleic acid binding.

Previous studies have demonstrated that the loop βA–βB (residues 25–44) within the P9-1 proteins of both MRCV and RBSDV comprises crucial RNA binding sites, despite lacking complete structural information (Figure 4B and C) (26,44). Our crystal structure of the NSP9^ΔC^ dimer reveals the formation of an antiparallel β-strand sheet (β-sheet II) in this specific region, supported by reliable electron densities. The dimeric arrangement of this β-sheet forms an awning-like structure, which creates a tunnel within the homodimer. To investigate the potential correlation between the nucleic acid binding capability of NSP9 and these structural features, we analyzed the amino acid composition and the surface electrostatic potential within this region. The study uncovered a notable concentration of positively charged amino acid residues in the tunnel (Figure 6A). Molecular docking was utilized to examine the interaction between the NSP9 dimer and single-stranded nucleic acid. The docking analysis results showed that the single-stranded nucleic acid was bound across the positively charged tunnel of the NSP9 dimer (Figure 6B), which was consistent with the putative nucleic acid binding site.

The arch-shaped structure contains six positively charged amino acids, specifically R9, K10, K12, K20, K23 and R28, which span residues 8 to 28 and form the tunnel. To investigate the impact of these amino acids on RNA binding, we performed mutagenesis and truncation experiments, along with EMSA. Analysis of the SEC elution profiles of various truncated protein samples showed that, despite exhibiting similar elution volumes to the wild-type NSP9^ΔC^ dimer, there was no significant alteration in the dimeric arrangement (Figure 6C). However, the deletion of residues 9–24 completely abolished the ability of NSP9^ΔC^ to bind nucleic acids (Figure 6D; Supplementary Figure S4A), thereby underscoring the vital importance of this particular region. In contrast, the disordered loop region between β6 and β7 may have less impact on nucleic acid binding capacity, as deletion of residues 76–117 did not significantly affect the nucleic acid binding affinity in EMSA experiments (Figure 6D). A subsequent analysis of single-point mutations in NSP9^ΔC^ further highlighted the crucial role of K20 in nucleic acid binding. Specifically, the K20E mutation significantly impaired the interaction between NSP9^ΔC^ and nucleic acids, while the S18A mutation had no discernible effect on nucleic acid binding (Figure 6E; Supplementary Figure S4B).

## Discussion

In this study, we focus on NSP9 to gain insights into the potential working mechanism of the membrane-less viral replication factory within the *Reovirales*. While prior investigations have attempted to unveil the working mechanism of the viral replication factory in rotavirus, the overall topography of the viral replication factory has been well characterized (2,7,20,48). However, there are still gaps in understanding the specific roles played by viral proteins within the viroplasm, as well as the unique translation regulation and genome assembly mechanisms of reoviruses in this context. Hence, a more in-depth discussion of the molecular basis of viroplasm proteins is imperative.

The recombinant overexpression of the full-length NSP9 protein resulted in the formation of oligomers, which subsequently led to the formation of disordered, amorphous, and irregular spherical structures. By using a combination of bioinformatics, biochemical, and structural methodologies, we discovered that the core part of NSP9 (NSP9^ΔC^) adopts a dimeric organization, sharing striking topological similarities with the P9-1 homodimers of MRCV, RBSDV and SRBSDV. NSP9 exhibits robust binding affinity to both single-stranded and double-stranded nucleic acids, regardless of their DNA or RNA origin, with a slight preference for single-stranded nucleic acids. Interestingly, the disordered region at the C-terminus of NSP9 mediates the higher-order oligomerization of the protein, yet it is dispensable for its nucleic acid binding capacity, contrasting with the findings on P9-1 (44). Furthermore, the dimeric form of NSP9 is essential for nucleic acid binding, which is attributed to the positively charged residues that form a tunnel during homodimer formation.

The assembly mechanism mediated by intrinsically disordered sequences is a ubiquitous trait among members of the order *Reovirales*, as evidenced by proteins such as P9-1 from MRCV, RBSDV and SRBSDV, where C-terminal disordered regions facilitate dimer-dimer interactions (24–26), and NSP2 from rotavirus, where C-terminal helices mediate interactions between two tetramers (49). Similarly, the N-terminal disordered region of reovirus σNS has been demonstrated to mediate helical oligomer formation (50). Despite the substantial variations in sequences and three-dimensional structures among these proteins, they exhibit remarkable similarities in molecular assembly, topological structures, and functionalities (50). This suggests the presence of common features among key viroplasm proteins within the order *Reovirales*. Interestingly, while the recently published AlphaFold3 algorithm predicted the monomer structure of NSP9^ΔC^ with high accuracy, it still failed to provide the correct interaction mode of the NSP9^ΔC^ dimer. Further inquiries are needed to elucidate the form of NSP9 assembly under physiological conditions and the potential biological functions of the C-terminal IDR beyond its role in molecular assembly.

We employed EMSA experiments, alongside structural analysis, molecular docking techniques, and mutagenesis validation, to uncover a potentially unusual mode of protein-nucleic acid interaction. As the protein concentration increased, distinct migration bands emerged sequentially, indicating that NSP9^ΔC^ is capable of forming varying configurations of protein-nucleic acid complexes. It is noteworthy that the gel migration pattern of the translation regulatory molecule NSP3 of rotavirus (51–53) and CPV NSP9 when interacting with nucleic acids exhibits a significant degree of similarity. This finding highlights a conserved protein-nucleic acid interaction mode in the *Reovirales* and demonstrates the functional similarity of certain NSPs in regulating translation processes.

Research on *Reovirales*, including the early transcription of CPVs (54), the development and operational mechanism of the viroplasm involving secondary transcription and translation processes (7,18,20), and the equimolar assembly of viral genomes (55), has attracted considerable attention from the scientific community. The genome of *Reovirales* consists of 9–12 segmented double-stranded RNA. The mRNA is characterized by a cap structure at the 5′ end and the absence of a polyadenylated tail at the 3′ end (51,56). Notably, both termini of all viral genomes contain short and conserved sequences that are identical across segments within a virus but differ between different viral species (57,58). The precise functions and roles of these conserved sequences remain elusive, although they are thought to serve as assembly signals that facilitate efficient genome packaging (55,59). Studies of the rotavirus have shown that the conserved sequence at the mRNA 3′ end serves as a translational element, which is recognized and bound by the non-structural protein NSP3 (51,60). Notably, the nucleic acid binding pattern of rotavirus NSP3, which has been demonstrated to interact with the last four conserved bases at the 3′ end of its mRNA, exhibits a similar occurrence of two consecutive shift bands in EMSA when bound to ssRNA (Supplementary Figure S5A and B). Additionally, NSP3 interacts with the translation initiation factor eIF4G, mimicking the function of PABP, to promote the translation of viral non-polyadenylated mRNA (52,61,62). However, the role of the conserved 5′ sequence remains unclear. In addition, studies on reoviruses suggest that besides the capped and non-polyadenylated primary mRNA, the secondary or late transcribed mRNA exists, which lacks both a polyadenylated tail and a 5′ cap structure (63–65). Interestingly, these late-transcribed mRNAs exhibit higher translation efficiency compared to primary mRNA (66), although the precise mechanisms underlying this observation remain unclear (65,67).

The EMSA results in our study reveal the potential of both CPV NSP9 and rotavirus NSP3 to form diverse complexes with single-stranded nucleic acids. The elucidated structure identified a positively charged tunnel surrounded by a compact NSP9^ΔC^ dimer as the nucleic acid binding channel, thereby suggesting a plausible interaction potentially between the protein and the nucleic acid from both termini. Based on current studies, we propose a hypothesis that NSP9 binds to viral mRNA in a unique manner (Supplementary Figure S6). Such a binding model is proposed by structural analysis, molecular docking, and mutagenesis validation. Despite numerous attempts, we have not been able to obtain structural information on the NSP9-nucleic acid complex, probably due to its significant dynamic nature. In addition, the precise mechanism and pattern of NSP9 recognition of nucleic acid sequences remain unclear. Given the structural similarity between NSP9 and P9-1, as well as the similar migration pattern of NSP9 and rotavirus NSP3 when binding to single-stranded nucleic acids, NSP9 may play a role in viral genome packaging and/or translational regulation, which could be closely associated with the viroplasm (26,53). Nevertheless, we hope that our research can provide valuable references and theoretical foundations for relevant researchers.

In all, it is believed that these questions about the precise assembly of non-structural proteins in *Reovirales* and the functional support of viral replication factories, as well as research related to the translational regulation process of viral mRNA, will become highly attractive spots and novel targets in the field of the *Reovirales* research.

## Supplementary Material

gkae803_Supplemental_File

## Data Availability

The atomic coordinates of *Bm*CPV1 NSP9^ΔC^ have been deposited in the Protein Data Bank under the code ID 8Y9K.
