# Supplementary material for: Crystal structure and nucleic acid binding mode of CPV NSP9: implications for viroplasm in Reovirales
Source: Nucleic Acids Res. 2024 Sep 17;52(18):11115–27. doi: 10.1093/nar/gkae803 (PMC11472163; doi:10.1093/nar/gkae803)
Supplement: gkae803_Supplemental_File [file gkae803_supplemental_file.pdf]

**Supplementary Information for**

# Crystal Structure and Nucleic Acid Binding Mode of CPV NSP9: Implications for Viroplasm in *Reovirales*

**Yeda Wang[1,*], Hangtian Guo[1,*], Yuhao Lu[1], Wanbin Yang [1], Tinghan Li[1] and Xiaoyun Ji[1,2]†**

[1]Department of Infectious Diseases, Nanjing Drum Tower Hospital, State Key Laboratory of Pharmaceutical Biotechnology, School of Life Sciences, Institute of Viruses and Infectious Diseases, Chemistry and Biomedicine Innovation Center (ChemBIC), Institute of Artificial Intelligence Biomedicine, Nanjing University, Nanjing, China.
[2]Engineering Research Center of Protein and Peptide Medicine, Ministry of Education, China.

[*]These authors contributed equally

To whom correspondence should be addressed. Tel: +86 25 89681657; Email: xiaoyun.ji@nju.edu.cn

This document includes:
Supplementary Figures S1 to S6

## SUPPLEMENTARY FIGURES

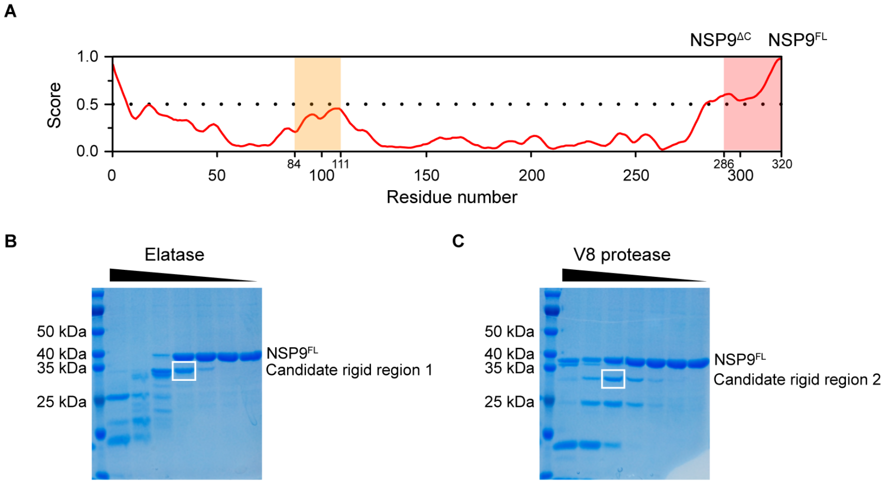


**Figure S1.** Molecular characteristics of NSP9 determined by bioinformatic prediction and restricted protease digestion assays. **(A)** The disordered score of NSP9, as calculated by IUPred3A, indicated the presence of an intrinsically disordered region at its C-terminus (residues 287–320), which is highlighted with a partially transparent red rectangle. The region of residues 84–111 highlighted with a partially transparent yellow rectangle was not resolved in the crystal diffraction data. **(B, C)** The restricted protease digestion assay was performed with elastase (B) and V8 protease (C), respectively. Two candidate rigid fragments were detected by SDS-PAGE and Coomassie blue staining, with a molecular weight of ~ 30–32 kDa, which is consistent with the bioinformatic prediction.

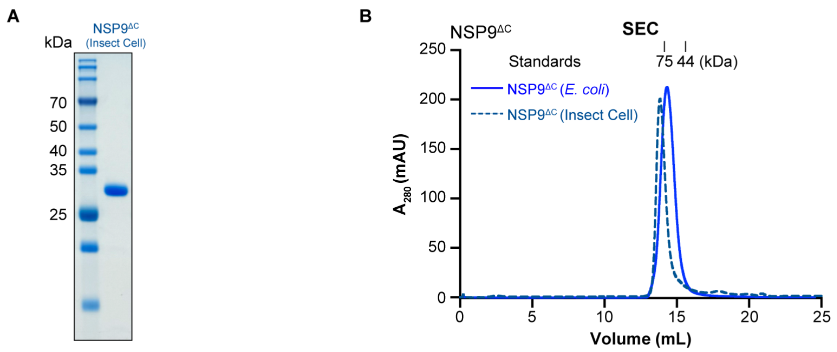


**Figure S2.** Analysis of NSP9$^{\Delta C}$ expressed in insect High Five cells. **(A)** The SDS-PAGE analysis of peak fractions of interest. **(B)** SEC analysis of purified NSP9$^{\Delta C}$ proteins expressed in either *E. coli* (solid line) or High Five cells (dashed line) using a Superdex 200 increase 10/300 column.

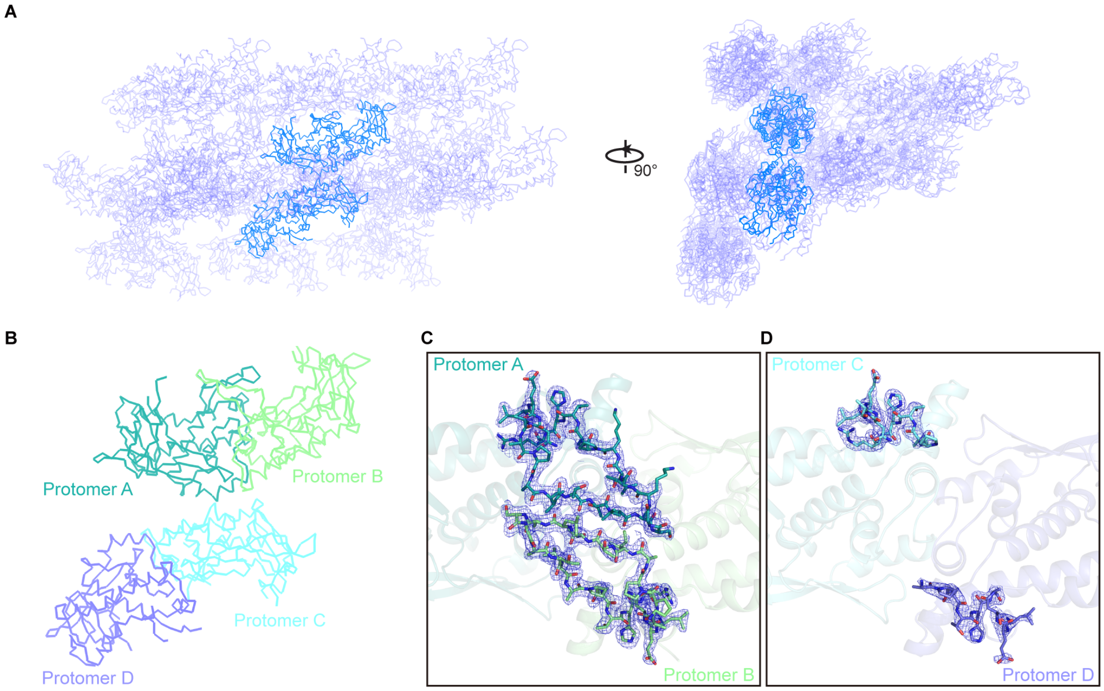


**Figure S3.** Crystal packing-mediated protein contacts in the NSP9$^{\Delta C}$ crystal structure. **(A)** The ribbon representation of four NSP9$^{\Delta C}$ protomers (colored royal blue) in an asymmetric unit surrounded by molecules (colored light purple) generated by the crystallographic symmetry. **(B)** Close-up view of four NSP9$^{\Delta C}$ protomers in an asymmetric unit, forming two homodimers. **(C, D)** Stereo view of a sample of the 2*mFo-DFc* electronic density map, which illustrates the interface of the awning-like structure between protomers A and B (C) and protomers C and D (D). Residues in this region are shown as stick representation. The density map is contoured at 1.0 σ.

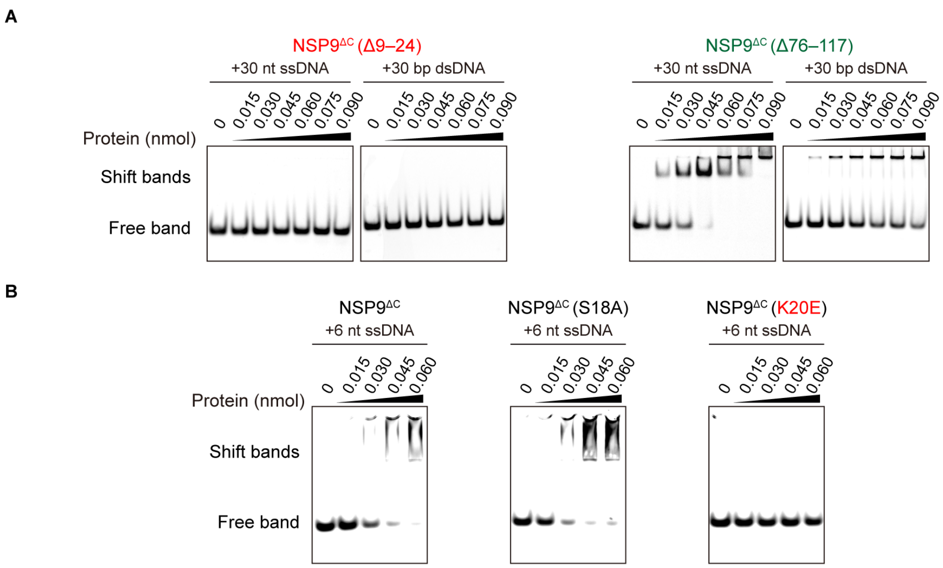


**Figure S4.** The EMSA results for NSP9 binding to ssDNA and dsDNA. (**A**) Comparison of EMSA results of NSP9$^{\Delta C}$ (Δ9–24) and NSP9$^{\Delta C}$ (Δ76–116) with either 30 nt ssDNA or dsDNA. (**B**) Comparison of EMSA results of NSP9$^{\Delta C}$, NSP9$^{\Delta C}$ (S18A) and NSP9$^{\Delta C}$ (K20E) with 6 nt ssDNA.

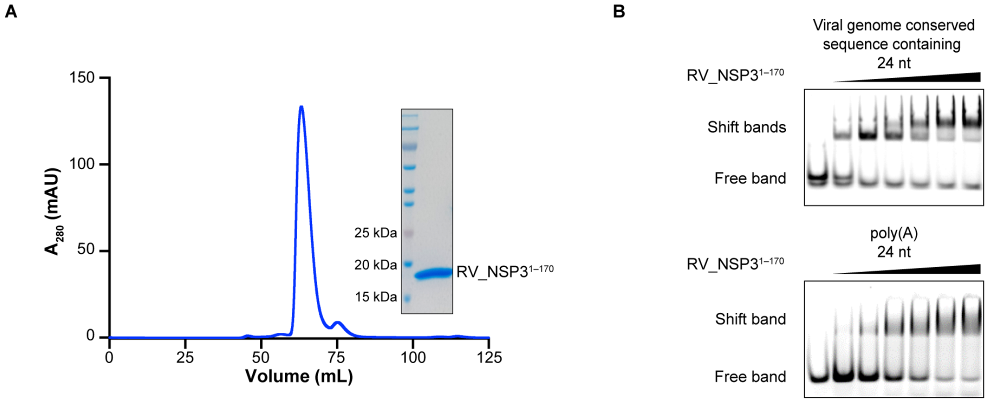


**Figure S5.** Protein purification and nucleic acid binding pattern of the RV_NSP3$^{1–170}$. (**A**) SEC analyses of purified RV_NSP3$^{1–170}$ using a HiLoad 16/600 Superdex 75 column and the SDS-PAGE analysis of peak fractions of interest. (**B**) EMSA results of RV_NSP3$^{1–170}$ with 0.03 nmol 5’ 6-FAM labeled 24 nt ssRNA. Two different sequences were used for immigration pattern detection, including a conserved sequence in viral genomes (GGCUUUUAAACGAAGGAUGUGACC) and poly(A), respectively.

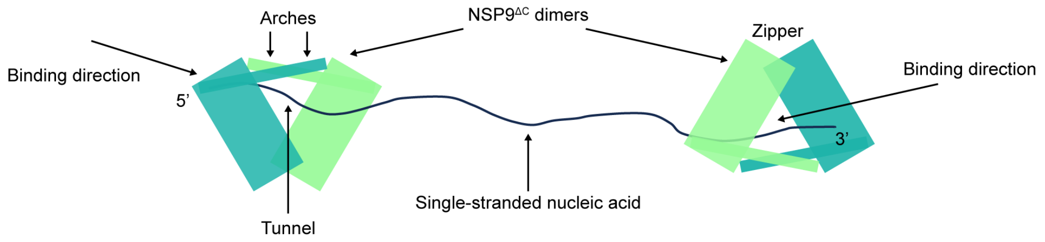


**Figure S6.** A proposed model for the binding of single-stranded nucleic acids by NSP9. The crystal structure of *Bm*CPV1 NSP9$^{\Delta C}$ reveals a dimeric arrangement. Arches from two NSP9$^{\Delta C}$ protomers interacted with each other to form an awning-like structure, while the other dimer interface served as a zipper-like structure. Two protomers in an NSP9$^{\Delta C}$ dimer together form a positively charged tunnel. NSP9 binds to two ends of single-stranded nucleic acid and forms different complexes in a protein concentration-dependent manner.
